# Stressed-Induced TMEM135 Protein Is Part of a Conserved Genetic Network Involved in Fat Storage and Longevity Regulation in *Caenorhabditis elegans*

**DOI:** 10.1371/journal.pone.0014228

**Published:** 2010-12-03

**Authors:** Vernat J. Exil, Daiana Silva Avila, Alexandre Benedetto, Elizabeth A. Exil, Margaret R. Adams, Catherine Au, Michael Aschner

**Affiliations:** 1 Thomas P. Graham Division of Pediatric Cardiology, Department of Pediatrics, Monroe Carell Jr. Children's Hospital at Vanderbilt University, Nashville, Tennessee, United States of America; 2 Department of Pediatrics, Center for Molecular Toxicology, Monroe Carell Jr. Children's Hospital, Nashville, Tennessee, United States of America; 3 London Centre for Nanotechnology, University College London, London, United Kingdom; Yonsei University, Republic of Korea

## Abstract

Disorders of mitochondrial fat metabolism lead to sudden death in infants and children. Although survival is possible, the underlying molecular mechanisms which enable this outcome have not yet been clearly identified. Here we describe a conserved genetic network linking disorders of mitochondrial fat metabolism in mice to mechanisms of fat storage and survival in *Caenorhabditis elegans* (*C. elegans*). We have previously documented a mouse model of mitochondrial very-long chain acyl-CoA dehydrogenase (VLCAD) deficiency.[1] We originally reported that the mice survived birth, but, upon exposure to cold and fasting stresses, these mice developed cardiac dysfunction, which greatly reduced survival. We used cDNA microarrays[2], [3], [4] to outline the induction of several markers of lipid metabolism in the heart at birth in surviving mice. We hypothesized that the induction of fat metabolism genes in the heart at birth is part of a regulatory feedback circuit that plays a critical role in survival.[1] The present study uses a dual approach employing both C57BL/6 mice and the nematode, *C. elegans*, to focus on TMEM135, a conserved protein which we have found to be upregulated 4.3 (±0.14)-fold in VLCAD-deficient mice at birth. Our studies have demonstrated that TMEM135 is highly expressed in mitochondria and in fat-loaded tissues in the mouse. Further, when fasting and cold stresses were introduced to mice, we observed 3.25 (±0.03)- and 8.2 (±0.31)- fold increases in TMEM135 expression in the heart, respectively. Additionally, we found that deletion of the *tmem135* orthologue in *C. elegans* caused a 41.8% (±2.8%) reduction in fat stores, a reduction in mitochondrial action potential and decreased longevity of the worm. In stark contrast, *C. elegans* transgenic animals overexpressing TMEM-135 exhibited increased longevity upon exposure to cold stress. Based on these results, we propose that TMEM135 integrates biological processes involving fat metabolism and energy expenditure in both the worm (invertebrates) and in mammalian organisms. The data obtained from our experiments suggest that TMEM135 is part of a regulatory circuit that plays a critical role in the survival of VLCAD-deficient mice and perhaps in other mitochondrial genetic defects of fat metabolism as well.

## Introduction

The very long-chain acyl-CoA dehydrogenase (VLCAD) enzyme catalyzes the first step in the mitochondrial fatty acid β-oxidation spiral. Fatty acids are the preferred substrates for ATP generation in the heart, skeletal muscles and high energy requiring tissues. It is well documented that mutation in the VLCAD gene leads to cardiomyopathy, skeletal myopathy, encephalopathy and sudden death in children and young adults.[5], [6], [7], [8], [9] Our previously published data on VLCAD-deficient mice showed that when the fasting and cold stresses were introduced, these animals exhibited decreased survival, recapitulating the clinical phenotypes of children with VLCAD deficiency.[10] In a previous study[1], we documented that, at birth, the surviving mice showed marked upregulation of both the peroxisome proliferator-activated receptorγ (PPARγ) coactivator-1 alpha (PGC-1α), which is a critical regulator of mitochondrial biogenesis, as well as acyl-CoA synthase, which is a facilitator of sarcolemmal fatty acid uptake.[1] The induction of these genes at birth further supported our hypothesis that the induction of these fat metabolism genes in the heart at birth is part of a feedback-regulated circuit that plays a critical role in survival in VLCAD deficiency. Another protein, TMEM135, which had not been previously characterized, was also found to be elevated in VLCAD-deficient mice on the first day of life. In this report, we sought to elucidate the role of TMEM135 in this hypothesized survival circuit. TMEM135 was first identified in a lung adenocarcinoma cell line cDNA library,[11] but a recently published paper suggests that TMEM135 might be a critical gene for adipogenesis and osteoblastogenesis.[12] Due to the fact that TMEM135 was found to be phylogenetically highly conserved, we hypothesized that the regulatory feedback circuit to which it belongs, as well as its function, might be equally conserved.

Recent papers have proposed the use of the nematode, *Caenorhabditis elegans* (*C. elegans*), for studies of fat metabolism, obesity and longevity.[13] Genes and pathways involved in coordinating the transport, sorting and storage of lipids, as well as fat metabolism, are well conserved in the worm and are critical for its long-term survival. Therefore, we decided to investigate the role of TMEM135 concurrently in the mouse and in the *C. elegans* model.

In this study, we also set out to replicate in *C. elegans* the metabolic model established in the mouse. We assessed the tissue distribution and sub-cellular localization of TMEM135 in the mouse and in the worm. We then used a *C. elegans* model to test the role of TMEM135 in modulating fat stores, longevity and cold sensitivity. This is the first study to demonstrate an association of TMEM135 with mitochondrial fat metabolism, and the first published data on a possible newly identified role for TMEM135 in enhancing fat storage, fat sorting and mitochondrial function in mammalian organisms and in the nematode.

With a focus on potential target molecules linking TMEM135 to a genetic network involved in fat biology and survival in the mouse, we also sought to determine if there exists a link between TMEM135 and insulin signaling within this genetic network. We deemed it particularly relevant to examine this possibility because it has been documented that insulin signaling plays a critical role in regulating fat stores and enhancing longevity in C. elegans.[14], [15], [16], [17], [18], [19], [20], [21] Given the serious human outcomes which result from VLCAD dehydrogenase enzyme deficiency, namely cardiac pathologies and sudden death, our studies may provide new insights into the molecular mechanisms that place newborn infants with these defects at a greater risk for sudden death.[22] There is a high degree of molecular heterogeneity in disease outcome and prognosis.[5] However, it remains to be determined whether molecular elements related to this putative feedback regulatory circuit account for the clinical spectrum in patients with identical genetic mutations. It is also uncertain whether the changes we have identified in protein expression patterns in the mice at birth[1] bear relevance to human patients. Nevertheless, the results of our studies definitively point to a critical role for TMEM135 in the biology of lipid storage and lipid utilization in both the mouse and the worm.

## Materials and Methods

### VLCAD knockout mice

#### Ethics Statement

VLCAD-deficient mice were generated as previously described.[1] Experiments involving these mice were undertaken in accordance with the guidelines and regulations set forth by the Animal Welfare Act, and the protocols were approved by the Vanderbilt University Institutional Animal Care and Use Committee (Approval ID numbers: M/00/535, M/03/070, and M/03/116).

### RNA extraction and northern blots

RNA extraction and northern blots were performed using mouse heart tissue, as previously described.[1] RNA (10 µg per sample lane) was resolved by electrophoresis in a formaldehyde-agarose gel and then transferred to a nitrocellulose membrane. We used 1 kb of the amino-terminal coding sequence of TMEM135 to generate [α- ^32^P] dCTP-labeled Random-Primer probes, employing the Prime-It II kit (Stratagene, La Jolla, CA).

### Protein isolation and immunoblotting

In the mouse, tissues were harvested representing the three VLCAD genotypes (^+/+^, ^+/−^, and ^−/−^) and were snap-frozen in liquid nitrogen, as previously described.[10] Frozen tissue stored at −70°C was used to assess the tissue distribution of TMEM135 in the heart, skeletal muscles, liver and brain of two-month-old mice. Western blot analysis was performed as previously described.[10] The expression of TMEM135 in the mouse was assessed with peptide-specific rabbit polyclonal antibodies against TMEM135 (1∶1000) (generated in immunized rabbits by Invitrogen antibody services, Carlsbad, CA). In *C. elegans*, the expression levels of DAF-16 (Santa Cruz Biotechnologies –sc 33738, 1∶5000), SKN-1/NRF2 (Abcam, ab 31163, 1∶5000), SOD3 (sc 67088, 1∶10000), AKT (sc 8312, 1∶1000), VLCAD (Strauss laboratory, rabbit polyclonal antibody) as previously published,[1] PGC-1α (sc 13067, 1∶1000), β- actin (sigma A 1978, 1∶10000) and ACS (from Dr. Jean Schaffer, Washington University, St. Louis, MO), as previously published [1] were assessed using rabbit polyclonal antibodies. Luminol-based detection was performed using horseradish peroxidase-conjugated anti-rabbit or anti-mouse IgG and the chemilunescence reagent, ECL (Amersham Pharmacia Biotech UK). Results are representative of at least three independent experiments.

### Sub-cellular fractionation

Sub-cellular fractionation of mouse hearts was performed as previously described.^11^ Hearts were harvested from euthanized animals in conformity with humane animal codes. Hearts were rinsed in phosphate-buffered saline (PBS), kept in ice-chilled PBS and then transported to a cold room (4°C).[23] To minimize protein degradation, initial experiments were performed at 4°C. Mitochondrial fractions were obtained from pellets saved by subsequent differential centrifugation. Sub-cellular organelles were then re-suspended in 200 µl of re-suspension buffer and snap-frozen in liquid nitrogen until use.

### Protein multiple alignment

Multiple-alignment of TMEM135 orthologues was generated with Clustal X 2.0.10 using default alignment parameters (Gonnet Matrix series) loaded with the following protein sequences retrieved from NCBI: NP_082619.3 for TMEM135 from *Mus musculus*, NP_075069.3 for TMEM135 from *Homo sapiens*, NP_001013918.1 for TMEM135 from *Rattus norvegicus*, NP_001082887.1 for TMEM135 from *Danio rerio*, NP_001085541.1 for TMEM135 from *Xenopus laevi*, NP_649803.1, NP_731251.1, NP_731250.1 for TMEM135-like protein from *Drosophila melanogaster*, and NP_508800.2 for TMEM-135 from *Caenorhabditis elegans*.

### *K02G10.3/tmem-135* cloning

The full *tmem-135* sequence from 850 bp upstream of the start codon down to the last codon was amplified by PCR with the TaKaRa LA Taq polymerase (Takara Bio Inc.) in an Eppendorf II thermocycler (Eppendorf) using the following primers: forward primer: 5′- tataggatccgaacaggcaattatggaagacc-3′, reverse primer: 5′- tataggtaccgcatattcaacaagtggcatgtaaagc -3′. It was then cloned into pPD95.75 (Andrew Fire Laboratory, Addgene Kit, please see http://www.addgene.org/docs/fire/andrew/datasheets.pdf) in frame with the green fluorescent protein (GFP) between the BamHI and KpnI restriction sites to generate the plasmid pMA0002.

### Handling of the worms and generation of *C. elegans* transgenic strains

*C. elegans* strains were handled and maintained at 20°C as previously described.24 The RB1443 strain carrying the 1.1 kb deletion *ok1646*, which impairs the K02G10.3 expression product, was obtained from the *C. elegans* consortium. The wild-type Bristol N2 strain and the BC15594 strain[25] expressing the GFP under the promoter of K02G10.3 (*k02g10.3::*GFP) were obtained from the *Caenorhabditis* Genetic Centre (CGC, Minnesota). The original RB1443 was outcrossed 4 times with N2 to remove undesirable genetic alterations resulting from ethyl-methanesulfonate (EMS) mutagenesis used to generate the original strain. We henceforth refer to the K02G10.3 expression product as TMEM-135, which stands for TMEM135-like protein. Homozygous mutant RB1443 animals will be further designated as *tmem-135*(*ok1646*) or *tmem* (*−/−*). We also generated eight independent full-length K02G10.3::GFP-expressing strains termed MAB123 (*mjaEx086*[K02G10.3::GFP; *rol-6*(*su1006*)]) to MAB130 (*mjaEx093*[K02G10.3::GFP; *rol-6*(*su1006*)]) by co-injection in young adult gonads pMA0002 at 25 ng/mL and pRF4 at 100 ng/mL. Single worm PCR was performed according to the procedures set forth by Barstead et al. (1991).[26] Fluorescent images for GFP were obtained by confocal microscopy.

### Life-span evaluation

For life-span evaluation, 20 worms from each group were transferred to NGM/FUDR plates, placed at two different temperatures (15 and 20°C) and scored every day. Delay of egg-laying in *C. elegans* hermaphrodites lead to accumulation of mixed-stage developing embryos in the uterus. These embryos eventually hatch and kill their parent. To prevent this undesirable effectin our lifespan assay, we used FUDR treatment of young adult worms to render them sterile. [27] These studies were performed for three independent cohorts, and the results are representative of triplicate experiments.

### Nile red staining and mitochondrial action potential imaging

The vital dye, Nile Red (5 H-benzo [α] phenoxazine-5-one, 9diethylamino), was used to visualize fat droplets in live worms as described in Greenspan et al.[28] Mitochondrial localization and distribution were assessed using MitoTracker Red (M22425 Invitrogen, Carlsberg, CA) according to procedures outlined as follows. Ten µM of MitoTracker Red in DMSO where diluted in 1 mL of M9 buffer (22 mM KH_2_PO_4_, 22 mM Na_2_HPO_4_, 85 mM NaCl, 1 mM MgSO_4_) in which synchronous L1 or young adult worms were incubated for 2 hours in the dark. Worms were then washed with M9 buffer in order to remove excess dye. Worms were subsequently transferred to NGM/OP50 plates for 2 hours to clear the intestinal tract of residual dye, after which they were paralyzed with a mixture of 0.01% levamisole/0.1% tricaine and mounted on 4% agarose pads for imaging. Fluorescence observations were performed with an epifluorescence microscope (Nikon Eclipse 80i, Nikon) equipped with a Lambda LS Xenon lamp (Sutter Instrument Company) and Nikon Plan Fluor 20x dry and Nikon Plan Apo 60×1.3 oil objectives. The microscope was coupled to a black-and-white camera (DS-Qi1Mc; Nikon) operated by the Nikon Elements AR3.0 software (NES AR3.0). Confocal images were acquired with a Zeiss LSM510 confocal microscope (Carl Zeiss Microimaging Inc.) equipped with a Plan-Apochromat 63x and a Plan-Neofluar 100× oil objectives with 1.4 and 1.3 apertures.

### Cold exposure

L1 worms were mounted on agarose pads, covered with cover-slips and left for 1 hour at either 4°C or at 20°C. Then, worms were brought to room temperature for 30 minutes prior to imaging. For each condition approximately 30 worms were observed under an epifluorescence microscope, and 5 were imaged by confocal microscopy.

### Data analysis and statistics

Data were plotted as means ± SEM. For GFP-fluorescence integrated intensity, northern and western blot densitometry analysis, two-tailed T-tests were used for single comparisons, while one-way analysis of variance (ANOVA) was used for multiple comparisons. Multiple comparisons were performed using the SPSS software package (SPSS). Differences were deemed significant for p<0.05. By convention, we noted *: p<0.05, **: p<0.01, ***: p<0.001.

## Results

### TMEM135 is overexpressed in the heart of VLCAD-deficient mice

**Figure 1:**
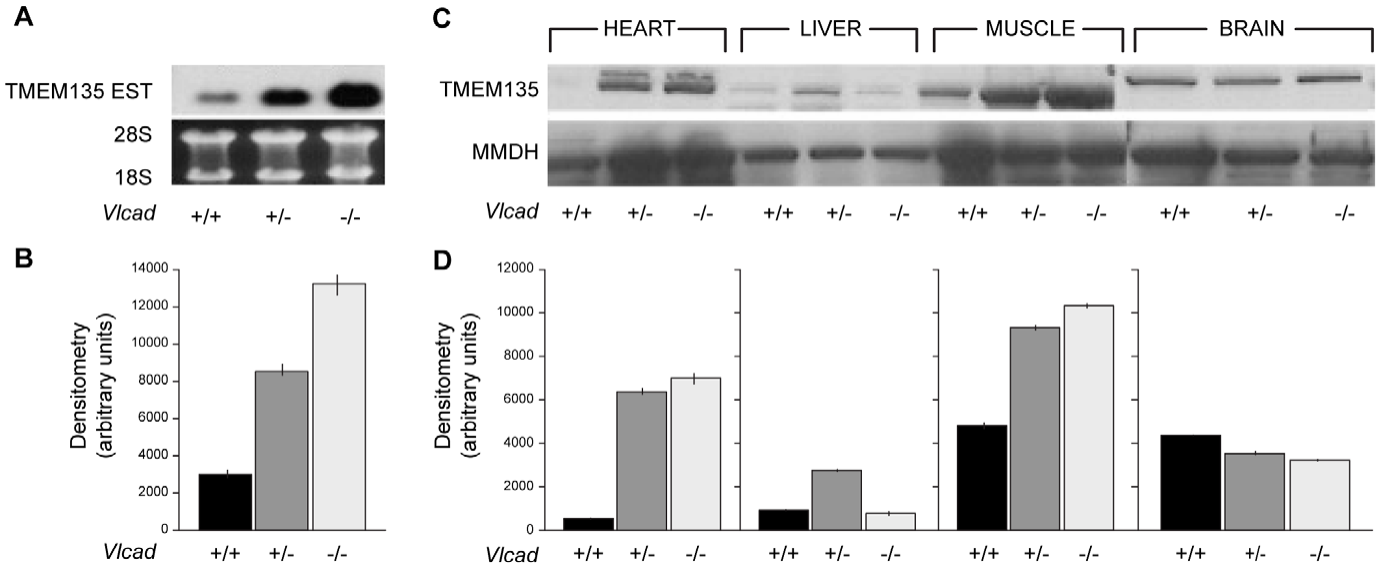
RNA and protein expression of TMEM135 in mouse tissues. (**A**) Northern blot analysis of TMEM135 transcripts in newborn hearts of mice deficient in the very long chain ACAD gene. (**B**) **B** is representative of quantification by densitometry of Northern blot in **A**. (**C**) Protein expression of TMEM135 in different mouse tissues. (**D**) Quantification by densitometry of western blots in **C**. N =  3 per group for wild-type control mice labeled (+/+) for VLCAD +/+, heterozygous mice (+/−) for VLCAD+/−, and null mutant mice (−/−) for VLCAD−/−.

We previously reported on a mouse model of VLCAD deficiency[1], [10], [23] that exhibited lipid accumulation in the heart as well as the marked induction of several indictors of lipid metabolism at birth, including PPARα, adipophilin and acyl-CoA synthase prior to any microscopic evidence of lipid droplets in the heart.[1] We performed cDNA microarrays of the VLCAD-deficient hearts[2], [3], [4], comparing one-day-old VLCAD-deficient hearts to wild-type controls. Detail results of the microarray studies are unpublished. These studies provided us with a previously uncharacterized expressed sequence tag (EST) that was markedly induced in hearts of VLCAD-deficient mice at birth. Results obtained from northern blot analysis showed that this novel EST was upregulated 4.3 (±0.14)-fold in hearts of VLCAD^−/−^ mice compared with those of VLCAD^+/+^ control mice (p<0.001). This EST was also upregulated 1.5-fold in the VLCAD^−/−^ mice compared with VLCAD^+/−^ mice (Fig. 1A and 1B). A sequence BLAST of this EST produced a full-length mouse sequence, including the 5′ and 3′ untranslated regions, which were found in the NCBI database. This EST corresponded to the uncharacterized gene, *tmem135.* Computer-generated analysis in protein databases revealed that this TMEM135 and its orthologues are membrane proteins, exhibiting 5 to 7 transmembrane domains, with multiple putative PKC phosphorylation and tyrosine kinase sites. The vertebrate sequences contain an epitope with isochemical homology to the 21 aa endothelin epitope and a putative nuclear hormone receptor motif “IRNLDDEL”.

### TMEM135 is induced in mice with mitochondrial fatty acid β-oxidation enzyme defects

To further characterize this novel protein, rabbit polyclonal antibodies were raised against the putative endothelin domain and against the carboxy terminal. We discovered, by western blot analysis, that the epitope was abundantly expressed in high energy-requiring tissues, such as the heart and the skeletal muscles of VLCAD +/− and VLCAD −/− mice (Fig. 1C and 1D). Subsequently, we tested the long-chain acyl-CoA dehydrogenase-deficient (LCAD) mouse hearts, which exhibit another form of fatty acid β-oxidation enzyme deficiency. TMEM135 was also found to be elevated in LCAD mutants (data not shown.) Both models, the VLCAD and LCAD mice, demonstrate an inability to metabolize very long- (C14–C18) and long- chain (C12–C14) fatty acids. [29], [30], [31] These mice are also sensitive to the cold and represent mouse models of human fatty acid β-oxidation defects. Based on our experimental data, we posited that a link exists between fatty acid metabolism and TMEM135 function in mitochondrial fatty acid β-oxidation.

### TMEM135 is highly conserved across animal species

**Figure 2:**
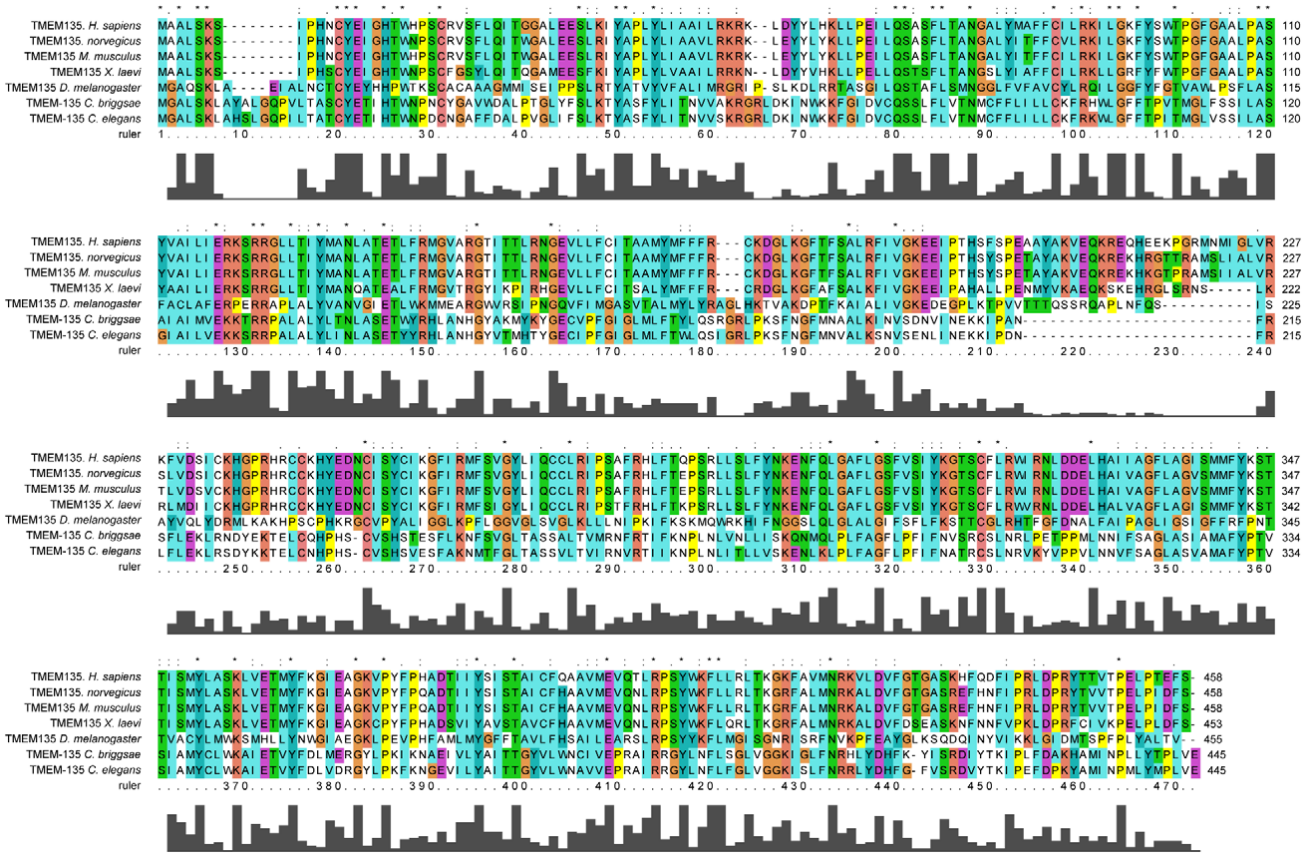
Comparison of sequence homology analysis of TMEM135 in different species. TMEM135 is well conserved across species.

Our computer search of the NCBI database revealed that the TMEM135 sequence is highly conserved across species from *C. elegans* to humans, as shown by protein multiple alignments (Fig. 2). The search further disclosed the existence of one short orthologue in each vertebrate species (data not shown), 3 full-length orthologues in Drosophila and a unique orthologue in *C. elegans*. As this protein was heretofore unnamed, we are labeling the *C. elegans* orthologue, TMEM-135 (TMEM135-like protein).

### TMEM-135 is ubiquitous and expressed in dot-like cytoplasmic organelles throughout development in *C. elegans*

**Figure 3:**
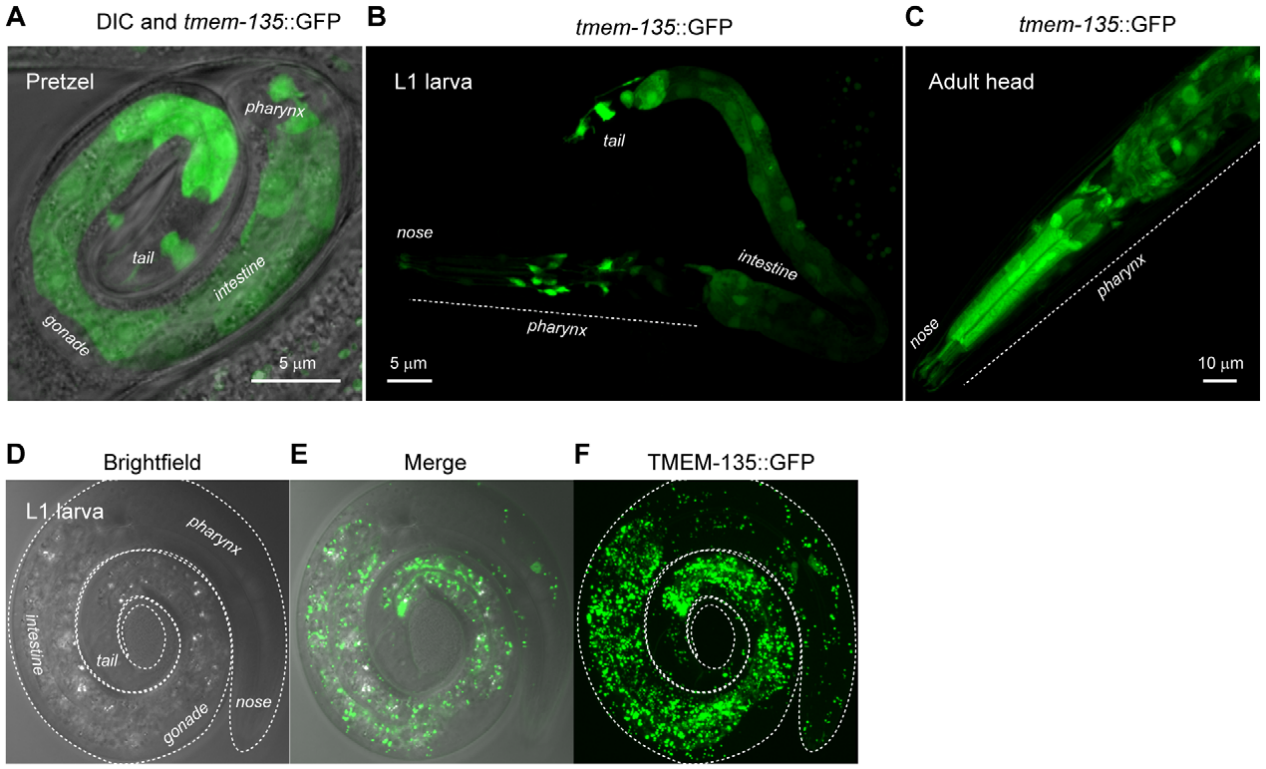
TMEM-135 distribution in *C. elegans*. Images in **A**, **B** and **C** were obtained using *tmem-135* promoter-driven GFP to assess *tmem-135* tissue expression. *tmem-135* was found to be ubiquitously expressed at all stages (A, B, C; data not shown). Apparent differences in expression pattern reflect the mosaic distribution of the transgene. (**A**) Shows *tmem-135* expression pattern in the pretzel embryo. (**B**) Shows *tmem-135* expression pattern in a L1 larva. (**C**) Shows *tmem-135* expression pattern in an adult worm. Images in **D**, **E** and **F** were obtained using a GFP-tagged full-length TMEM135 to observe TMEM135 sub-cellular localization. Panels **D**, **E** and **F** reveal that TMEM135 localizes to rounded sub-cellular organelles of 0.2–0.5 microns in L1 larvae, which are particularly abundant in the intestine.

Given that this protein was highly conserved across species (Fig. 2), we used *C. elegans* to further investigate its role in the hypothesized metabolic feedback mechanism that regulates survival under stress. We obtained a *promoter::*GFP-expressing strain and cloned the full-length open reading frame in phase with GFP to study the TMEM-135 expression pattern and sub-cellular localization. In the *tmem-135 promoter*::GFP-expressing strain, as well as in the eight independent TMEM-135::GFP-expressing strains, we consistently observed an ubiquitous expression from the pretzel embryo to the adult stage (Fig. 3A–C), despite mozaicism due to the extra-chromosomal array. More specifically, *tmem-135* expression was more strongly expressed in the lower intestine (Fig. 3A and 3B) and in the pharynx (Fig. 3A and 3C). TMEM-135::GFP showed a high level of expression throughout the entire length of the intestine, as well as in all epithelia (Fig. 3D–F). The sub-cellular localization of this protein appeared to correspond to dot-like cytoplasmic structures of 0.2 to 1 micron in L1 worms.

### TMEM135 expression shows an association with fat droplets and mitochondria in mice and worms

**Figure 4:**
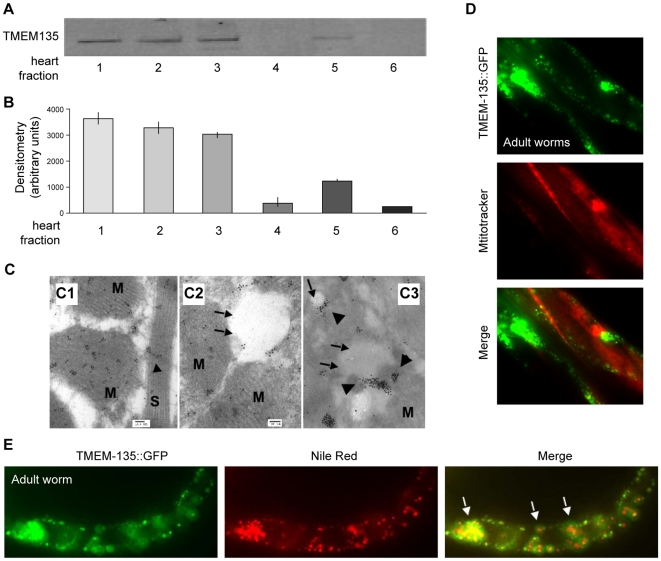
TMEM135 localizes to fat droplets in the mouse and in *C. elegans* and to mitochondria close to fat droplets in the mouse. Experiments in A, B and C were done in mouse tissue. (**A**) Western blot to assess protein expression of TMEM135 in sub-cellular fractions of the mouse heart. TMEM135 specific antibody, 1∶1000. Lane1: total protein, lane 2: nuclear fraction, lane 3: mitochondrial fraction, lane 4: microsomal fraction, lane 5: a low speed sediment containing intercalated discs sediment and microsomes enriched in heart sarcoplasmic reticulum, lane 6: cytosol. (**B**) Quantification by densitometry of western blot in **A**. **C1**, **C2** and **C3** are representative of Transmitted Immuno-Electron microscopy in mouse heart tissue. (**C1**) Immunogold staining showing TMEM135 mostly in the mitochondria and along the Z-line in the sarcomere. M =  mitochondria, S =  sarcomere. **C2 and C3** show accumulation of the TMEM135 epitope in the surrounding of lipid droplets in mouse hearts. M =  mitochondria, Arrows =  fat droplets, Arrowheads =  TMEM135 gold stain. (**D**) Is representative of TMEM135::GFP staining with Mitotracker Red, showing no co-localization of TMEM135 and the mitochondria in the worm. (**E**) Represents Nile red staining in the TMEM135 over-expressing animals. This figure shows co-localization of the GFP and fat droplets in the worm, as indicated by the arrows. Experiments in this section were performed 3 or 4 times.

We next determined the compartment to which the TMEM135 protein localizes. We first used sub-cellular fractionation to assess the sub-cellular localization of TMEM135 in the mouse heart. Our results revealed a strong enrichment of TMEM135 in the mitochondrial and nuclear fractions (Fig. 4A and 4B). Immuno-transmission-electron microscopy (ITEM) confirmed the mitochondrial localization and revealed an even stronger staining around fat droplets and in mitochondria surrounding those in the mouse heart (Fig. 4C). Some nuclear staining was observed (data not shown), but with much less consistency, a result possibly due to non-specific staining or because the putative nuclear localization identified may be highly regulated (i.e., seen under specific conditions). We next tested to determine whether, in *C. elegans* expressing TMEM-135::GFP, stained organelles would correspond to mitochondria or fat droplets. We used the lipophilic dyes, Nile Red and Mitotracker Red, to assess the co-localization of TMEM-135 with fat droplets or mitochondria, respectively. We observed no co-localization of TMEM-135::GFP and MitoTracker Red (mitochondrial) (Fig. 4D) in the worm, whereas TMEM-135::GFP and Nile Red displayed very strong co-localization (Fig. 4E). Together, the mouse and worm data provide strong evidence that the TMEM135 protein is associated with fat droplets, supporting our hypothesis that TMEM135 plays a role in fat metabolism.

### TMEM135 is upregulated upon fasting and cold exposure in worms and in mice

**Figure 5:**
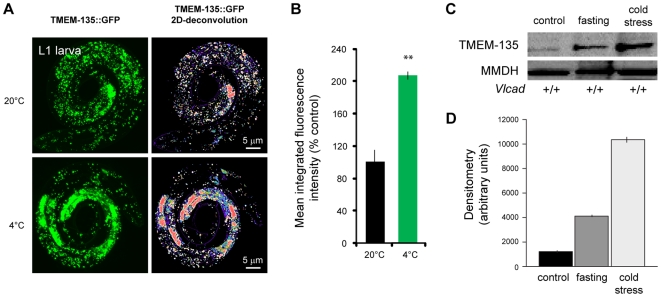
TMEM135 is elevated with the stresses of cold and fasting in the worm and in C57BL/6 mice. Figures **A** and **B** are representative experiments in the worm. Fig. **A** shows the induction of TMEM135 with cold stress (4°C) in the worm, and Fig. **B** is the quantification of average fluorescence levels exemplified in Fig. **A**. Values in these experiments are expressed as relative fluorescence intensity in mean ± SEM. Figures **C** and **D** were experiments done using C57BL/6 mice. The animals were subjected to overnight fasting and were exposed to the cold for 2 hours, as previously published[10]. (**C**) Western blots of TMEM135 expression with fasting and cold stresses. (**D**) Quantification by densitometry of the western blots shown in C, N = 3 per group.

Based on our findings that TMEM135 is highly expressed in VLCAD mice at birth (when lipid stores are strongly mobilized), and given a potential role for TMEM135 in fatty acid oxidation, we hypothesized that TMEM135 protein levels would be upregulated upon physiological stresses such as cold exposure and fasting (conditions in which fat stores and fat utilization are required). Thus, we introduced these stressors as functional tests to demonstrate the role of TMEM135 in fat metabolism. We found that TMEM135::GFP was upregulated upon 1 hour of cold exposure in L1 worms (Fig 5A and 5B). We observed a 2 fold increase in the average integrated-fluorescence signal upon cold exposure in the worm (arbitrary unit of 100±14 in control animals vs 213±5.5 in cold-exposed worms, p<0.01). Moreover, although TMEM135 expression was not highly expressed in normal mouse heart tissue, TMEM135 was upregulated 3.25 (±0.03)-fold upon fasting (p<0.05) and 8.2 (±0.31)-fold upon cold exposure (p<0.02) (Fig. 5C and 5D). These results confirm that TMEM135 plays a conserved role in stress conditions that require fat mobilization.

### Loss of TMEM-135 leads to reduction of fat stores in the worm

**Figure 6:**
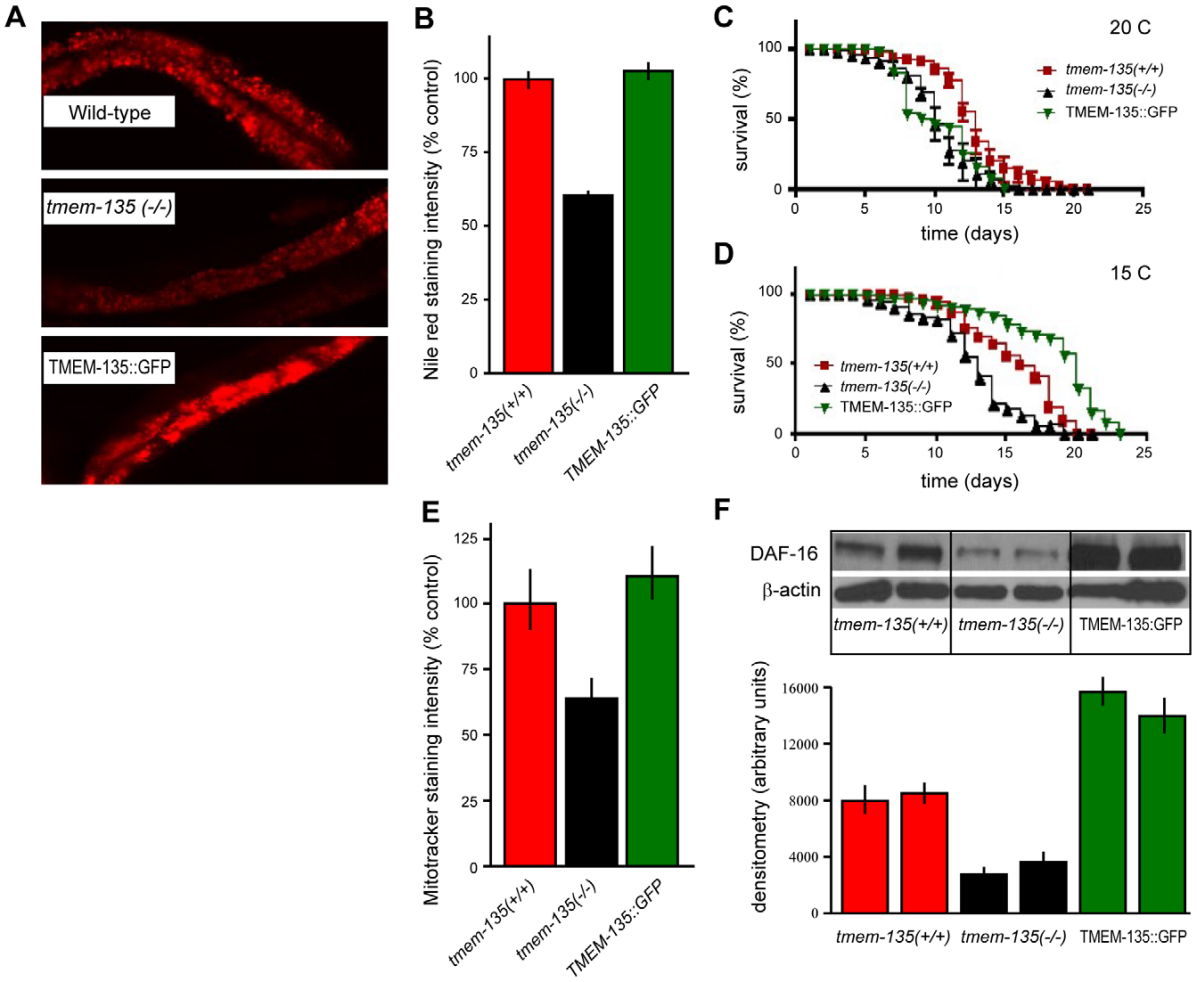
Nile Red staining and MitoTracker Red staining, survival differences and DAF-16 levels among the three *C. elegans* strains. Fig. **6A** is representative staining with Nile Red in the worm. (**B**) Represents semi-quantitative assessment of Nile Red staining intensity, N = 4 per group. (**C**) Represents survival analysis among the three *C. elegans* strains at 20°C. (**D**) Represents survival analysis among the three *C. elegans* strains at 15°C. Wild-type  =  controls shown in red, *tmem 135*(−*/*−) * =  tmem135*-deleted animals shown in black, TMEM135::GFP =  *C. elegans* animal overexpressing TMEM135 shown in green. (**E**) Quantitative assessment of MitoTracker Red fluorescence. (**F**) Western blot analysis and quantification of DAF-16 levels in the three *C. elegans* strains, N = 6 per group, values are mean ± SEM.

Next, we hypothesized that TMEM135 may be involved in the modulation of fat stores in the worm. To test this hypothesis, we obtained the *C. elegans* deletion mutant, *tmem135*(*ok1646*). Then, using Nile Red staining, we compared fat stores in wild-type, TMEM-135::GFP-overexpressing and *tmem135*(*ok1646*) mutant worms and found that the *tmem135*(*ok1646*) mutants exhibited significantly weaker Nile Red staining compared with the other strains. We observed a 41.8% (±2.8%) reduction in Nile Red staining in *tmem135*(*ok1646*) animals compared with N2 controls (p<0.0002) (Fig. 6A and 6B). When compared with wild-type controls, TMEM-135::GFP-expressing worms did not show any significant difference in Nile Red staining. Additionally, pharyngeal pumping did not differ among the three genotypes (data not shown), suggesting that the reduction in fat stores in *tmem135*(*ok1646*) worms was not due to reduced feeding. Hence, these results support a direct role for TMEM-135 in the regulation of fat stores in the worm.

### TMEM-135 deletion reduces worm longevity while overexpression increases it at 15°C

Based on the assumption that fat stores are regulated by TMEM135, we hypothesized that changes in TMEM135 levels in worms may affect their longevity. To test this hypothesis, we measured differences in longevity among the three strains (wild-type controls, *tmem135* (*ok1646*) mutant and TMEM135::GFP-overexpressing worms) at different temperatures. Longevity experiments revealed that the *tmem135* (*ok1646*) mutants lived for a significantly shorter time than wild-type worms, both at 20°C (normal growth conditions) and 15°C (low temperature), while TMEM135::GFP-overexpressing worms lived for a significantly longer time than wild-type worms at 15°C (Fig. 6C and 6D). The experiments conducted at 15°C showed that, in growth conditions requiring increased fat stores (low temperature), reduced TMEM135 expression was associated with a shorter life-span, while increased TMEM135 expression led to an increased life-span. These results strongly suggest that TMEM135 plays a pivotal role in the network that regulates the fat stores required for survival under cold and normal conditions in the worm.

### TMEM135 deletion is associated with reduced mitochondrial potential in the worm

To better characterize survival differences in the cold, we used the mitochondrial potential sensing ability of the Mitotracker Red staining and compared fluorescence in the three *C. elegans* genotypes. We found a 40.7% (±8.0%) reduction in Red fluorescence in *tmem135* (*ok1646*) animals compared with wild type-controls (p<0.02; Fig. 6E). These results indicate that mitochondrial activity is impaired in *tmem135* (*ok1646*) worms, likely contributing to cold sensitivity in the *tmem135^−/−^* animals. However, changes in mitochondrial activity cannot explain increased longevity in the cold for TMEM135::GFP mutants, as we did not observe a significant difference in the amount of Mitotracker Red fluorescence between TMEM-135::GFP-overexpressing animals and N2 controls.

### TMEM-135 modulates FoxO/DAF16 expression levels in *C. elegans*

FoxO/DAF-16 is well known as an important requirement for ensuring normal longevity in the worm.[15], [16] Given the impact of TMEM-135 deletion on the worm lifespan, we wondered if the loss of TMEM-135 expression in *tmem-135*(*ok1646*) mutants would be associated with a reduction of DAF-16 expression. Indeed, western blot analysis showed a significant 60.7% (±3.3%) reduction in FoxO/DAF-16 expression in *tmem-135*(*ok1646*) worms compared with wild-type controls (p<0.006) (Fig. 6F). Furthermore, TMEM-135::GFP overexpression led to a 2.0-fold up-regulation of FoxO/DAF-16 levels compared with wild-type controls (p<0.002) (Fig. 6F). Based on these results, we have concluded that TMEM135 is normally involved directly or indirectly in FoxO/DAF-16/regulation. Additional studies are needed to test whether DAF-16 down-regulation accounts for the reduction in fat stores, mitochondrial membrane potential and life-span in *tmem135* (*ok1646*) worms.

### TMEM135 deletion does not lead to protein expression changes in the Nrf-2/SKN-1 antioxidant pathway

**Figure 7:**
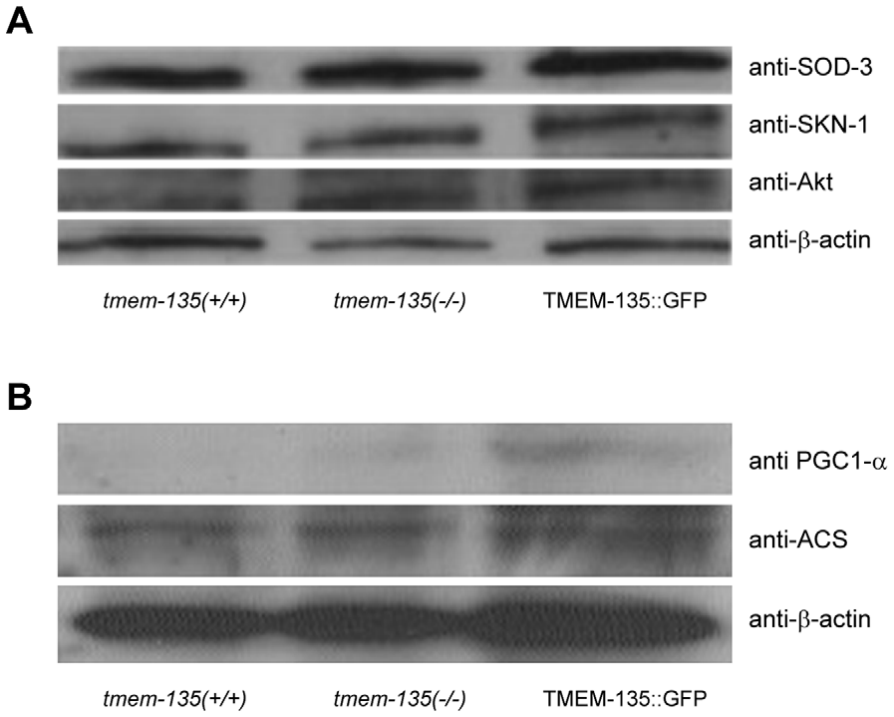
Western blot analysis of possible downstream targets of TMEM135. **7A** is representative western blot for NRF2/SKN1 targets. **7B** is representative western blot analysis of fatty acid metabolism target genes. N = 3 in each group. *tmem135*(*−/−*) for the tmem135-deletion strain, and TMEM135::GFP for the overexpressing strain. N = 3 in each group.

Longevity regulation in the worm implicates the conserved Insulin/IGF-1 pathway which inhibits the two major longevity-promoting transcription factors, FoxO/DAF-16 and Nrf2/SKN-1. Since TMEM-135 expression was found to affect DAF-16 expression levels (Fig. 6F), we hypothesized that it could similarly impact Nrf2/SKN-1 expression. To test this hypothesis, we sought to determine whether *tmem135*(*ok1646*) mutant and TMEM-135::GFP-overexpressing worms would exhibit an alteration in Nrf2/SKN-1 levels. When compared with wild-type worms, we found no differences (Fig. 7). We recognize, however, that Nrf2/SKN-1 expression levels are not necessarily informative, since Nrf2/SKN-1 activity regulation mainly relies on its translocation to the nucleus where it exerts its effect. To gain access to Nrf2/SKN-1 activity, we therefore quantified, by western blot analysis, the protein levels of a classic downstream target of Nrf2/SKN-1, superoxide dismutase, *sod-3*.[32] We found that the antioxidant enzyme level was unchanged in the *tmem135* (*ok1646*) and TMEM135::GFP animals (Fig. 7). These results indicate that TMEM135 does not interfere with the Nrf-2/SKN-1 antioxidant pathway.

### TMEM-135-dependent alteration in DAF-16 levels is independent of AKT expression

FoxO/DAF-16 is a known downstream target of the Insulin/IGF-1 pathway via inhibition by the AKT-1+AKT-2+SGK-1 complex. As DAF-16 levels were affected by TMEM-135, and TMEM-135 was shown to regulate fat stores (Fig. 6A and 6B), an implication of TMEM-135 in the Insulin/IGF-1 was deemed likely. Therefore, we measured AKT levels by western blot analysis in *tmem-135*(*ok1646*), TMEM-135::GFP-overexpressing and wild-type worms. We found no significant differences in AKT expression levels between the three genetic backgrounds (Fig. 7), although we can't rule out a possible involvement of AKT or the classical Insulin/IGF-1 pathway in TMEM135-dependent modulation of DAF-16 expression.

## Discussion

In this study, we utilized comparative biology between the mouse and the nematode, *C. elegans*, to identify and characterize a novel protein associated with mitochondrial fatty acid metabolism, fat stores, longevity, and energy expenditure. We hypothesized that this novel protein plays a critical role in a feedback regulatory circuit involved in survival in VLCAD deficiency. Our results also led us to undertake a systematic study linking TMEM135 to fat storage and longevity in *C. elegans*. A novel finding of these pursuits is that TMEM135 promotes longevity in the worm. The TMEM135 transgene rescued the cold stress phenotype and increased longevity under cold temperature conditions, while TMEM135 deletion in the worm led to decreased longevity at all experimental temperatures. These findings in both the worm and in the mouse support our original hypothesis that TMEM135 plays a critical role for survival in response to cold stress, as well as in the metabolic circuitry involving mitochondrial function and energy expenditure. In humans, mitochondrial fatty acid oxidation defects are associated with a variety of clinical phenotypes.[5], [33] We and others have hypothesized that there are co-founding elements involved in the heterogeneity of clinical phenotypes and outcome,[1], [5], [34], [35] although an age-associated phenotype-genotype correlation has been described.[36] Given that cold and fasting stresses are often lethal for mice with defects in the fatty acid β-oxidation pathway,[10], [30] our results suggest a role for TMEM135 among the plausible candidate genes involved in modulating overall survival and/or stress-associated clinical phenotypes. Future studies must address whether findings in the worm will prove beneficial for elucidating the possible role of TMEM135 in human patients with these metabolic gene defects.

Another novel finding in this report is the discovery that TMEM135's pro-longevity function seems linked to DAF-16 modulation, since we were able to show that TMEM135 deletion or TMEM135 overexpression *in vivo* correlates with changes in DAF-16 levels. Functional copies of DAF-16, an orthologue of the mammalian FOX3a,[16], [17] which, in both humans and worms, determines longevity, also regulates fat storage in worms.[19] The DAF-2 insulin receptor, IP3-kinase, AKT1 and AKT2, phosphorylate Daf16/FOXO to inhibit its downstream targets. The inhibition of DAF-2 signaling leads to the activation of DAF-16 through dephosphorylation, as well as the translocation of DAF-16 to the nucleus to activate transcription. The translocation of DAF-16 between the cytosol and the nucleus through phosphorylation and dephosphorylation is a dynamic process, serving as a true switch for nutrient sensing, metabolic substrate utilization and stress resistance. [37] There are several other pathways known to regulate DAF-16. Among them, the targets of rapamycin (TOR) kinase and c-Jun N-terminal kinase (JNK) have both been shown to contribute to various cellular responses and to aging, possibly in parallel or downstream of DAF-16.[38] JNK was recently found to be a positive regulator of DAF-16. [39] It appears that TMEM135 modulates DAF-16 levels, fat stores and longevity in a pathway either upstream or in parallel to the insulin-signaling pathway. One hypothesis is that DAF-16 and TMEM135 act on an identical pathway to modulate survival. This notion is strengthened by the fact that the lifespan was shortened and DAF-16 and fat stores were found to be reduced in the *tmem-135*-null worms. Ogg et al. reported that fat accumulation assayed with Sudan Black is reduced in *daf-16*(*mgDF50*) mutants. This group hypothesized that DAF-16 acts as a repressor of metabolic genes that mediate energy usage and that the loss of function of these proteins causes a metabolic shift leading to a reduction in fat mobility. [17] A metabolic shift to accumulate fat forms the basis for life-span extension in the worm. In agreement with this theory, it has also been shown that *daf-2* and *age-1* mutants (critical components of the insulin signaling pathway) demonstrate increased longevity and increased energy stores [21]. On the other hand, the fact that TMEM135 seems to regulate both DAF-16 levels (a regulator of lipid stores) and the lack of alteration in AKT and SOD levels raises the possibility that TMEM135 and DAF-16 cooperate in this metabolic shift through mechanisms which may be independent of the insulin signaling pathway.

We recognize that there are limitations to our study. Our analysis does not address how a loss of function in genes that affect mitochondrial fatty acid metabolism leads to the activation of TMEM135. Additionally, in the present study, we have not addressed the mechanism(s) leading to DAF-16 dysregulation in the absence of TMEM135. Our observation that the TMEM135 transgene increases DAF-16 levels but does not lead to an increase in survival at 20°C, provides a basis for further studies. However, our results corroborate previously published data showing that the DAF-16 transgene does not lead to increased longevity, which is in contrast to the increase seen with the reduction in DAF-2 function. Future studies in the worm addressing targets of TMEM135 might include the acyl-CoA synthase (ACS), the acyl-CoA dehydrogenases and PGC-1 α. Although levels of ACS, VLCAD and PGC-1α were not changed in the TMEM135-deficient animals, we did find that TMEM135::GFP-overexpressing mutants displayed elevated levels of ACS, VLCAD and PGC-1α (Fig. 7). These results might be related to DAF-16 overexpression, as DAF-16/FoxO targets include these fatty acid-associated genes.[40] FoxO transcription factors have been shown to enhance fatty acid uptake and oxidation, leading to increased levels of genes of fatty acid metabolism (i.e. ACS) in C2 C12 cells.[41] FoxO is also known to decrease the expression of Acyl-CoA carboxylase, leading to reduced levels of malonyl-CoA, which is an inhibitor of fatty acid oxidation.[41] In addition, PGC-1α has been shown to be a master regulator of mitochondrial biogenesis,[42] as well as the transcription co-factor providing homeostatic control of cellular ATP. [43] Given that the overexpression of TMEM135 in the GFP::TMEM135 worm led to increase survival in the cold, and overexpression of ACS, VLCAD and PGC-1α, it is certainly conceivable that TMEM135 is involved in facilitated fatty acid flux, mitochondrial biogenesis, and energy expenditures. Our findings place TMEM135 among the plausible candidate genes involved in our hypothesized feedback regulatory circuit, a circuit critical to enhancing the rate of survival in fatty acid oxidation-deficient mice. Among the three *C. elegans* strains tested, we did not detect changes in SOD3, NRF2 or AKT (Fig. 7). Thus, we determined that TMEM135 regulates fat stores and longevity in the worm by modulating DAF-16 levels and fat metabolism genes, without causing apparent changes in oxidative stress response genes.

In summary, we have demonstrated that TMEM135 is a key regulator of longevity and fat stores in *C. elegans*. Both in the mouse and in the worm, TMEM135 seems to be critical for enabling adaptation to energy challenges (i.e. cold and fasting stresses). Data obtained from studies in both the mouse and the worm suggests that TMEM135 might function as a nutrient sensor and a metabolic regulator gene. We propose that, in the mouse, TMEM135 is part of a feedback regulatory circuit involving the induction of several fat metabolic genes at birth that may aid in survival. Based on the results of our recent experiments which have tested this hypothesis, it could be anticipated that disturbances in levels of any of the genes involved in this feedback circuit may be trigger outcomes that lead to energy deprivation, as well as sensitivity to cold and fasting stresses, which would be consistent with the clinical phenotypes currently observed in human infants with disease-causing genetic mutations in the mitochondrial fatty acid oxidation pathway. Extrapolating from the results of our studies, we note that, in cases of fatty acid oxidation deficiency, the fact that there are compensatory genes expressed at birth, and before the appearance of disease in the mouse, it is possible that these molecules might serve as therapeutic targets for restoring the metabolic profile necessary for adequate energy balance, appropriate fatty acid flux and sufficient metabolic stress response. Given that mitochondrial energy metabolism, fat sorting and storage are also critical for other aspects of cell function, our findings suggest that TMEM135 may also be a critical factor in a variety of other chronic illnesses.
